# Citrulline protects human retinal pigment epithelium from hydrogen peroxide and iron/ascorbate induced damages

**DOI:** 10.1111/jcmm.17294

**Published:** 2022-04-23

**Authors:** Chervin Hassel, Morgane Couchet, Nathalie Jacquemot, Christelle Blavignac, Cécile Loï, Christophe Moinard, David Cia

**Affiliations:** ^1^ Université Clermont Auvergne INSERM U1107 NEURO‐DOL, Laboratoire de Biophysique Neurosensorielle Clermont‐Ferrand France; ^2^ Université Grenoble‐Alpes INSERM U1055, Laboratoire de Bioénergétique Fondamentale et Appliquée Grenoble France; ^3^ 27006 Université Clermont Auvergne Centre Imagerie Cellulaire Santé Clermont‐Ferrand France; ^4^ CITRAGE Boissy St Léger France

**Keywords:** ARPE‐19 (human RPE cell line), citrulline, hydrogen peroxide, iron/ascorbate, oxidative stress

## Abstract

Oxidative stress plays an important role in the ageing of the retina and in the pathogenesis of retinal diseases such as age‐related macular degeneration (ARMD). Hydrogen peroxide is a reactive oxygen species generated by the photo‐excited lipofuscin that accumulates during ageing in the retinal pigment epithelium (RPE), and the age‐related accumulation of lipofuscin is associated with ARMD. Iron also accumulates with age in the RPE that may contribute to ARMD as an important source of oxidative stress. The aim of this work was to investigate the effects of L‐Citrulline (CIT), a naturally occurring amino acid with known antioxidant properties, on oxidative stressed cultured RPE cells. Human RPE (ARPE‐19) cells were exposed to hydrogen peroxide (H_2_O_2_) or iron/ascorbate (I/A) for 4 h, either in the presence of CIT or after 24 h of pretreatment. Here, we show that supplementation with CIT protects ARPE‐19 cells against H_2_O_2_ and I/A. CIT improves cell metabolic activity, decreases ROS production, limits lipid peroxidation, reduces cell death and attenuates IL‐8 secretion. Our study evidences that CIT is able to protect human RPE cells from oxidative damage and suggests potential protective effect for the treatment of retinal diseases associated with oxidative stress.

## 1 INTRODUCTION

Oxidative stress appears to play an important role during ageing of the retina and in the pathophysiology of retinal diseases, such as age‐related macular degeneration (ARMD).^1^, ^2^, ^3^ The retinal pigment epithelium (RPE), localized between the choroid and the neural retina, is particularly vulnerable to oxidative damage caused by reactive oxygen species (ROS).^4^ Hydrogen peroxide (H_2_O_2_) is a ROS generated during RPE phagocytosis of photoreceptor outer segments^5^, ^6^ and during light irradiation of melanin present in the RPE.^7^ This oxidant is also produced by the photo‐excited lipofuscin that accumulates with age in the RPE, and its accumulation is associated with ARMD.^8^ Also, it has been reported that iron levels increase in RPE during ageing and that age‐dependent iron accumulation is accelerated in patients with ARMD.^9^, ^10^, ^11^ Additionally, accumulation of iron can be toxic to the RPE. Indeed, the increase of intracellular ferrous iron produces hydroxyl and lipid alkoxyl radicals through the Fenton reaction, leading to lipid peroxidation and protein oxidation.^12^, ^13^ Moreover, intracellular iron can interact with bisretinoid lipofuscin in RPE to promote cell damage.^14^ Evidence for the involvement of oxidative stress and free radical damage in RPE degeneration during ageing and ARMD are also reported from studies showing that oral intake of antioxidants could reduce the risk of developing ARMD.^15^ In addition, inflammation is implicated in the molecular mechanisms of ARMD pathogenesis, leading to RPE damage. The systemic and ocular levels of some pro‐inflammatory and pro‐angiogenic cytokines, such as Interleukin 8 (IL‐8), have been correlated with the incidence of ARMD.^16^ Increased expression of IL‐8 induced by oxidative stress is one of the earliest events of inflammation which could explain, at least in part, the inflammatory events involved in ARMD.^17^

L‐Citrulline (CIT), a naturally occurring amino acid, could be a good candidate for the prevention or treatment of retinal pathologies associated with oxidative stress. CIT has already won its spurs as antioxidant since it is a powerful hydroxyl radical scavenger.^18^ CIT has also been reported to protect against lipid peroxidation and circulating lipoprotein oxidation, as well as to decrease protein carbonylation in muscle and brain.^19^, ^20^, ^21^, ^22^ Moreover, studies evidenced that CIT is beneficial in neurological pathologies associated with oxidative stress^23^, ^24^, ^25^ and that this amino acid could be protective in the neurodegenerative process associated with ageing.^26^ Finally, this amino acid is a precursor of arginine and nitric oxide, and therefore plays a key role at the cardiovascular and cerebral levels.^27^ CIT is naturally synthesized by enterocytes from arginine or glutamine, and once released into the bloodstream escapes splanchnic sequestration and reaches the kidney where it is converted to arginine.^25^ This amino acid is almost absent from the diet, with the exception of watermelon (citrullus vulgaris) where it is present in high concentrations. It is also present in smaller amounts in cucumbers, pumpkins, melons and squashes. Furthermore, this amino acid is safe, well tolerated and has excellent bioavailability (80% of ingested CIT is found in the systemic blood circulation), as it has been largely demonstrated in both young adults and elderly subjects.^28^, ^29^, ^30^, ^31^ For these reasons, it seems that CIT could be a therapeutic strategy for the prevention/treatment of retinal pathologies. CIT could easily spread in the retina, due to its very good bioavailability, and its involvement in other retinal function (vasodilation of retinal arterioles) has recently been shown after oral administration in rats.^32^

The aim of the present study was to investigate the effects of CIT on oxidative stressed RPE cells. We have shown that CIT can protect human RPE cells from damage induced by H_2_O_2_ or iron/ascorbate. To our knowledge, this is the first study describing the effects of CIT against oxidative stress in RPE cells.

## 2 MATERIALS AND METHODS

### 2.1 Chemicals and reagents

Citrulline (CIT) was kindly provided by CITRAGE^®^ Company. Dulbecco's modified eagle medium (DMEM) F‐12 nutrient mixture (Ham) was from Gibco, foetal bovine serum (FBS) from Gibco and penicillin/streptomycin from Gibco. 3‐(4,5‐dimethylthiazol‐2‐yl)‐2,5‐diphenyl tetrazolium bromide (MTT), hydrogen peroxide (H_2_O_2_), iron (II) sulphate (FeSO_4_), sodium L‐ascorbate and catalase assay kit were purchased from Sigma. 2′,7′‐dichlorofluorescin diacetate (DCFDA)—cellular reactive oxygen species detection assay kit was obtained from Abcam. Boron‐dipyrromethene (Bodipy) C11 probe was from Life Technologies. FITC annexin V apoptosis detection kit with propidium iodide and IL‐8 assay kit were obtained from Biolegend. Lactate dehydrogenase (LDH) cytotoxicity assay kit was from Thermo Scientific.

### 2.2 RPE cell cultures

Adult human retinal pigment epithelial (ARPE‐19) cells were maintained in DMEM/F12 supplemented with 10% (v/v) FBS and 1% (v/v) antibiotics. Cells were cultured in 96‐well or 24‐well plates depending on the experiments. They were seeded at 100,000 cells/ml and grown at 37°C and 5% CO_2_ until they reached confluence (3 days). Confluent cells were treated with Citrulline (CIT, 1–400 mM) and hydrogen peroxide (H_2_O_2_, 0.6 mM) or iron/ascorbate (I/A, 7.5 mM/0.3 M). H_2_O_2_ and I/A were used to induce oxidative stress and to mediate lipid peroxidation.

### 2.3 Cell treatments

Pre‐ and co‐treatments were carried out with CIT as follows. In co‐treatment, cell cultures received a medium containing the oxidant in the presence of CIT for 4 h. In pretreatment, cultures first received a medium containing CIT for 24 h; the medium was then removed and replaced with a fresh culture medium containing the oxidant for 4 h.

### 2.4 Cell metabolic activity

Cell metabolic activity was determined by the 3‐(4,5‐dimethylthiazol‐2‐yl)‐2,5‐diphenyl tetrazolium bromide (MTT) assay. After treatment with the oxidant and/or CIT, RPE cells were rinsed in phosphate buffer saline (PBS) and incubated for 2 h with fresh culture medium containing 0.5 mg/ml MTT. During this incubation time, mitochondrial dehydrogenases of living cells reduced MTT to purple formazan. Cells were then rinsed in PBS and the insoluble purple formazan product was dissolved with dimethyl sulfoxide, forming a coloured solution. After centrifugation at 2000 *g* for 5 min, the absorbance of the supernatants, proportional to the number of living cells, was read at 570 nm with a microplate reader. The results are expressed as the percentage of control condition representing 100% of viability (cells incubated in normal medium only = 100% of absorbance).

### 2.5 ROS production

Reactive oxygen species (ROS) were measured in RPE cells using the probe 2′,7′‐dichlorofluorescin diacetate (DCFDA). The cell permeant reagent DCFDA is deacetylated by cellular esterases to dichlorofluorescein (DCFH), which can be oxidized by ROS into the fluorophore 2′,7′‐dichlorofluorescein (DCF). First, RPE cells were seeded on white, opaque‐bottomed 96‐well plates. On Day 3, the media were removed and the cells were washed with 1× Buffer (supplied with the kit) and incubated for 45 min at 37°C in 1× Buffer containing 25 µM DCFDA. The cells were then washed with 1× Buffer and treated with the oxidant and/or CIT in 1× Buffer for 4 h at 37°C. DCF production was measured by fluorescence spectroscopy with excitation wavelength at 485 nm and emission wavelength at 535 nm. The results are expressed as the percentage of control group (100% of fluorescence intensity).

### 2.6 Lipid peroxidation

Lipid peroxidation was determined by flow cytometry using the Boron‐dipyrromethene (Bodipy) C11 probe. The Bodipy is a lipophilic fluorescent dye that incorporates into biological membranes and responds to oxidation with a spectral emission shift from red to green. First, cultured RPE cells were incubated with Bodipy (5 µM) for 30 min at 37°C in DMEM/F12 1% FBS. Then, cells were treated with the oxidant and/or CIT and were analysed by a BD‐LSRII flow cytometer with FACSDiva Software (BD Biosciences) at the Cellular Health Imaging Center of Clermont Auvergne University. The results are expressed as the percentage of oxidized cells (green‐C11‐BODIPY_581/591_ stained cells) and non‐oxidized cells (red‐C11‐BODIPY_581/591_ stained cells).

### 2.7 Cell death

Cell death was quantified by flow cytometry using FITC annexin V (Ann) and propidium iodide (PI). After treatment with the oxidant and/or CIT, RPE cells were detached with trypsin‐EDTA, resuspended in fresh culture medium and stained with Ann (0.05 µg/ml) and PI (2.5 µg/ml). After incubating for 10 min at room temperature in the dark, cells were analysed by a BD‐LSRII flow cytometer with FACSDiva Software (BD Biosciences) at the Cellular Health Imaging Center of Clermont Auvergne University. Cells were sorted according to their size (FSC) and granularity (SSC), and cell states were identified as follows: living cells (Ann−, PI−), early apoptotic cells (Ann+, PI−) and late apoptotic/necrotic cells (Ann+, PI+). The results are expressed as the percentage of living cells, early apoptotic cells and late apoptotic/necrotic cells.

### 2.8 LDH release

Lactate dehydrogenase (LDH) released from injured RPE cells into the culture medium was quantified by a coupled enzymatic reaction in which LDH catalyses the conversion of lactate to pyruvate via NAD+ reduction to NADH. Diaphorase then uses NADH to reduce a tetrazolium salt to a red formazan product. After treatment with the oxidant and/or CIT, the supernatants were collected and mixed with reaction mixture. Following incubation in the dark for 30 min at room temperature, the absorbance, proportional to the quantity of LDH released into the culture medium, was determined at 490 nm using a microplate reader. The results are expressed in units of absorbance (LDH levels).

### 2.9 Interleukin‐8 production

Interleukin‐8 (IL‐8) released in the medium was determined by enzyme‐linked immunosorbent assay (ELISA). After treatment of RPE cells with the oxidant and/or CIT, the media were removed and replaced with a fresh culture medium. Following 24 h incubation, the supernatants were harvested for measuring IL‐8 by ELISA. Briefly, capture antibody was diluted in coating buffer and applied to a 96‐well plate overnight. Next, cell culture medium samples were added to each well and incubated for 2 h at room temperature after which the detection antibody was added for 1 h. After washing, avidin‐horseradish peroxidase was added to each well and left to incubate for 30 min at room temperature. The substrate solution was then added to each well for 30 min in the dark. Finally, a stop solution was added to inhibit the reaction, and the absorbance was read at 450 nm. The results are expressed as pg of IL‐8 per ml of medium.

### 2.10 Statistical analysis

The results correspond to the means ± SEM of *n* independent experiments. In each experiment, all conditions were done at least in triplicate. Statistical analysis was performed using Student's *t*‐test: *p < 0.05, **p < 0.01, ***p < 0.001.

## 3 RESULTS

### 3.1 Cytotoxic effects of CIT in RPE cells

**FIGURE 1:**
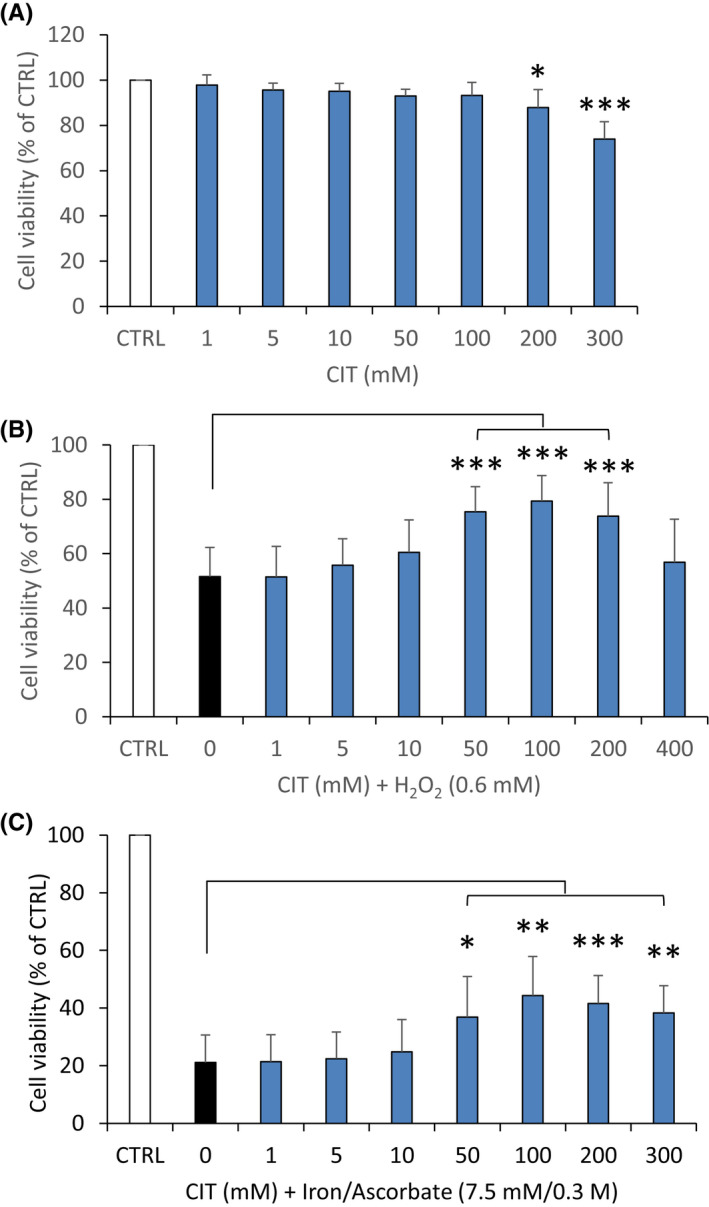
Effects of CIT on cell metabolic activity in oxidative stressed RPE cells. (A) Cytotoxicity of CIT in RPE cells. ARPE‐19 cells were incubated with CIT (1–300 mM) for 24 h, and cell metabolic activity was determined by MTT assay. Data are presented as means ± SEM (*n* = 8 independent experiments, each condition at least in triplicate). *p < 0.05 and ***p < 0.001, *t*‐test. (B) Effects of CIT in hydrogen peroxide (H_2_O_2_) stressed RPE cells. ARPE‐19 cells were treated with H_2_O_2_ 0.6 mM in the presence of CIT (1–400 mM) for 4 h, and cell metabolic activity was determined by MTT assay. Data are presented as means ± SEM (*n* = 10 independent experiments, each condition at least in triplicate). Treatment of RPE cells with H_2_O_2_ causes a decrease in cell viability, whereas co‐treatment with CIT 50, 100 and 200 mM significantly reduces this decrease. ***p < 0.001 vs. H_2_O_2_‐exposed cells without CIT treatment, *t*‐test. (C) Effects of CIT in iron/ascorbate (I/A) stressed RPE cells. ARPE‐19 cells were pretreated with CIT (1–300 mM) for 24 h and then exposed to I/A (7.5 mM/0.3 M) for 4 h. Cell metabolic activity was determined using MTT. Data are presented as means ± SEM (*n* = 9 independent experiments, each condition at least in triplicate). Treatment of RPE cells with I/A induces a decrease in cell viability, whereas pretreatment with CIT 50, 100, 200 and 300 mM significantly reduces this decrease. *p < 0.05, **p < 0.01 and ***p < 0.001 vs. I/A‐exposed cells without CIT pretreatment, *t*‐test. All the results are expressed as the percentage of control condition (CTRL = 100% of cell viability). CIT, L‐Citrulline; RPE, retinal pigment epithelium

We first assessed the toxicity of CIT on human RPE cells. For this purpose, cell cultures were incubated with several concentrations of CIT for 24 h, and cell viability was measured using MTT. As shown in Figure 1A, CIT did not affect metabolic activity of ARPE‐19 cells from 1 mM to 100 mM, but exhibited significant decreases from 200 mM. Therefore, the results show that CIT is relatively safe for RPE cells at concentrations up to 100 mM.

### 3.2 CIT improves cell metabolic activity in oxidative stressed RPE cells

To determine whether CIT can protect RPE cells from oxidative damage, we examined the effect of CIT against oxidative stress induced by H_2_O_2_. In a first set of experiments, cell cultures were incubated with H_2_O_2_ in the presence of CIT at different concentrations for 4 h, and cell metabolic activity was measured using MTT. As shown in Figure 1B, treatment of RPE cells with H_2_O_2_ 0.6 mM caused a significant decrease in cell viability (52 ± 11%), whereas co‐treatment with CIT 50, 100 and 200 mM significantly reduced this decrease (75 ± 9%, 79 ± 9% and 74 ± 12% of cell viability, respectively). In another set of experiments, cell cultures were pretreated with increasing concentrations of CIT for 24 h, washed and exposed to H_2_O_2_ 0.6 mM for 4 h. Pretreatment of the cells had no protective effect against H_2_O_2_, at any of the CIT concentrations tested (data not shown).

We also examined the effect of CIT against damage induced by iron/ascorbate (I/A). The combination of iron and ascorbate triggers a Fenton reaction with formation of hydroxyl radicals, which causes lipid peroxidation, membrane damage and cell death. As shown in Figure 1C, exposure of RPE cells to I/A 7.5 mM/0.3 M for 4 h led to a significant decrease in cell viability (21 ± 9%), whereas pretreatment of the cells with CIT 50, 100, 200 and 300 mM for 24 h significantly reduced this decrease (37 ± 14%, 44 ± 14%, 42 ± 10% and 38 ± 9%, respectively). Conversely, co‐treatment of the cells with CIT and I/A did not improve metabolic activity, at any of the CIT concentrations tested (data not shown).

Thus, CIT is able to reduce the toxicity of H_2_O_2_ and I/A in RPE cells, as shown by MTT assay. CIT is effective against H_2_O_2_ in co‐treatment, while it is effective against I/A in pretreatment. As we did not observe any improvement against H_2_O_2_ with CIT in pretreatment, only co‐treatments were carried out in the following experiments. Likewise, as no protection was observed against I/A with CIT in co‐treatment, only pretreatments were performed in the following experiments.

### 3.3 CIT decreases H_2_O_2_‐induced ROS production in RPE cells

**FIGURE 2:**
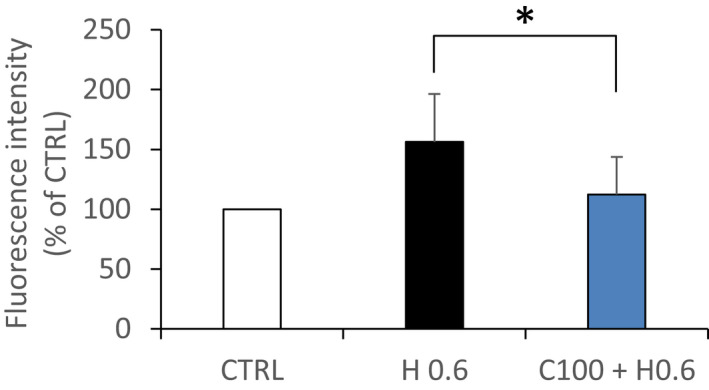
Effect of CIT on reactive oxygen species (ROS) production in hydrogen peroxide (H_2_O_2_) stressed RPE cells. ARPE‐19 cells were first incubated with DCFDA for 30 min and then treated with H_2_O_2_ 0.6 mM (H 0.6) in the presence of CIT 100 mM (C100) for 4 h. Fluorescent intensity was measured and expressed as percentage of untreated cells (CTRL). Data are presented as means ± SEM (*n* = 8 independent experiments, each condition at least in triplicate). Exposure of RPE cells to H_2_O_2_ increases intracellular ROS levels, whereas co‐treatment with CIT significantly reduces this increase. *p < 0.05 vs. H_2_O_2_‐exposed cells without CIT treatment, *t*‐test. CIT, L‐Citrulline; RPE, retinal pigment epithelium

We investigated whether CIT could counteract intracellular production of ROS induced by H_2_O_2_. As shown in Figure 2, exposure of RPE cells to H_2_O_2_ 0.6 mM for 4 h increased intracellular ROS levels by 56% compared to the untreated cells (CTRL). On opposite, co‐treatment with CIT 100 mM decreased ROS production by 29 ± 4% in comparison with cells treated with H_2_O_2_ alone.

### 3.4 CIT limits lipid peroxidation in oxidative stressed RPE cells

**FIGURE 3:**
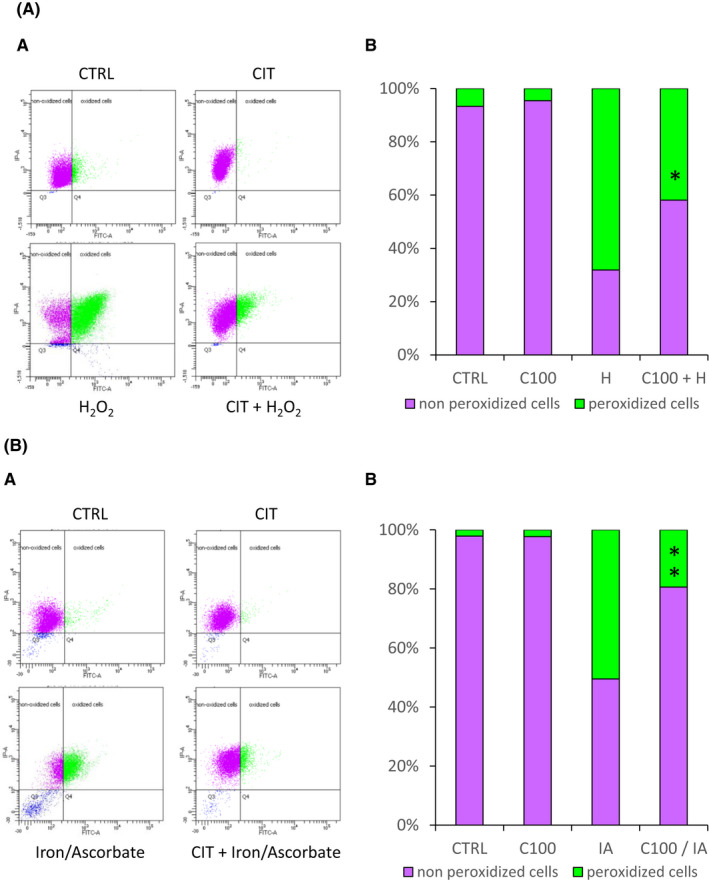
Effects of CIT on lipid peroxidation in oxidative stressed RPE cells. (A) Effects of CIT in hydrogen peroxide (H_2_O_2_)‐stressed RPE cells. ARPE‐19 cells were treated with H_2_O_2_ 0.6 mM (H) in the presence of CIT 100 mM (C100) for 4 h. Lipid peroxidation was quantified by flow cytometry using the Boron‐dipyrromethene (Bodipy) C11 probe. (A) Representative flow cytometry analysis showing non‐oxidized cells (red‐C11‐BODIPY_581/591_ stained cells, Q1) and oxidized cells (green‐C11‐BODIPY_581/591_ stained cells, Q2). (B) The results are expressed as the percentage of oxidized cells and non‐oxidized cells, and are presented as means ± SEM (*n* = 6 independent experiments). Treatment of RPE cells with H_2_O_2_ increases lipid peroxidation, whereas co‐treatment with CIT significantly reduces this increase. *p < 0.05 vs. H_2_O_2_‐exposed cells without CIT treatment, *t*‐test. (B) Effects of CIT in iron/ascorbate (I/A)‐stressed RPE cells. ARPE‐19 cells were pretreated with CIT 100 mM (C100) for 24 h and then exposed to I/A (7.5 mM/0.3 M) for 4 h. Lipid peroxidation was quantified by flow cytometry using the Boron‐dipyrromethene (Bodipy) C11 probe. (A) Representative flow cytometry analysis showing non‐oxidized cells (red‐C11‐BODIPY_581/591_ stained cells, Q1) and oxidized cells (green‐C11‐BODIPY_581/591_ stained cells, Q2). (B) The results are expressed as the percentage of oxidized cells and non‐oxidized cells, and are presented as means ± SEM (*n* = 5 independent experiments). Treatment of RPE cells with iron/ascorbate increases lipid peroxidation, whereas pretreatment with CIT significantly reduces this increase. **p < 0.01 vs. cells exposed to I/A without CIT treatment, *t*‐test. CIT, L‐Citrulline; RPE, retinal pigment epithelium

To evaluate the effect of CIT on lipid peroxidation induced by H_2_O_2_, RPE cell cultures were treated with the oxidant in the presence of CIT for 4 h, and lipid peroxidation was analysed by flow cytometry using Bodipy. As shown in Figure 3A, exposure of RPE cells to H_2_O_2_ 0.6 mM led to 67 ± 14% of oxidized cells (green staining), whereas co‐incubation with CIT 100 mM significantly decreased the percentage of stained cells (42 ± 20%) compared to cells treated with H_2_O_2_ alone.

We also examined the effect of CIT against lipid peroxidation induced by I/A. Cell cultures were pretreated with CIT for 24 h and then exposed to I/A for 4 h, and lipid peroxidation was quantified by flow cytometry. As shown in Figure 3B, treatment of RPE cells with I/A 7.5 mM/0.3 M resulted in 46 ± 11% of green‐stained cells. A pretreatment of the cells with CIT 100 mM before exposure to I/A significantly reduced the proportion of stained cells (19 ± 7%).

### 3.5 CIT reduces cell death in oxidative stressed RPE cells

**FIGURE 4:**
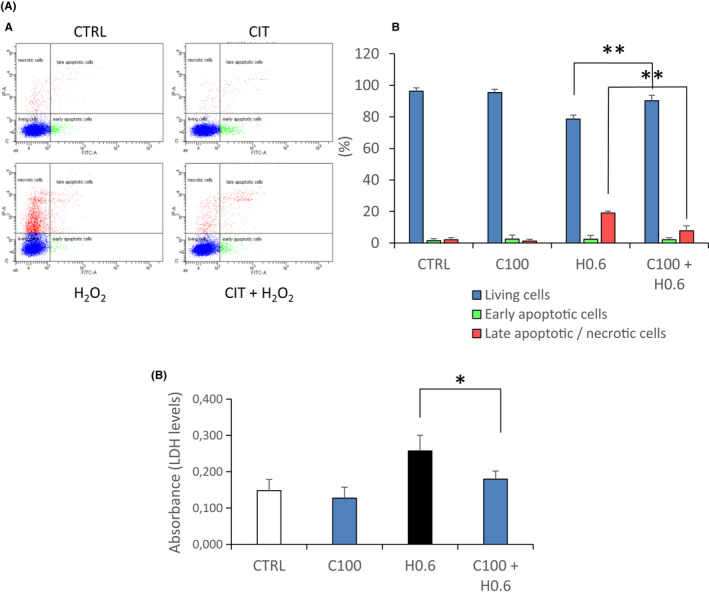
Effects of CIT on cell death in hydrogen peroxide (H_2_O_2_) stressed RPE cells. (A) Effects of CIT on cell death. ARPE‐19 cells were treated with H_2_O_2_ 0.6 mM (H 0.6) in the presence of CIT 100 mM (C100) for 4 h. Cell death was quantified by flow cytometry using annexin V‐FITC (Ann) and propidium iodide (PI). The cell populations were identified as follows: living cells (Ann−, PI−), early apoptotic cells (Ann+, PI−) and late apoptotic/necrotic cells (Ann+, PI+). (A) Representative flow cytometry analysis showing living cells (Q1), early apoptotic cells (Q2) and late apoptotic (Q3)/necrotic cells (Q4). (B) The results are expressed as the percentage of total cells and presented as means ± SEM (*n* = 4 independent experiments). Treatment of RPE cells with H_2_O_2_ increases cell death, mainly late apoptosis/necrosis, whereas co‐treatment with CIT significantly reduces this increase. **p < 0.01 vs. H_2_O_2_‐exposed cells without CIT treatment, *t*‐test. (B) Effects of CIT on lactate dehydrogenase (LDH) release. ARPE‐19 cells were treated with H_2_O_2_ 0.6 mM (H 0.6) in the presence of CIT 100 mM (C100) for 4 h. LDH released from injured cells into the culture medium was determined by LDH assay. The results are expressed in absorbance units and presented as means ± SEM (*n* = 4 independent experiments). Treatment of RPE cells with H_2_O_2_ induces LDH release from RPE cells, whereas co‐treatment with CIT significantly reduces this release. *p < 0.05 vs. H_2_O_2_‐exposed cells without CIT treatment, *t*‐test. CIT, L‐Citrulline; RPE, retinal pigment epithelium

To evaluate the protective effect of CIT on H_2_O_2_‐induced cell death, RPE cell cultures were incubated with H_2_O_2_ in the presence of CIT for 4 h, and cell death was quantified by flow cytometry using annexin V FITC and propidium iodide. As shown in Figure 4A, treatment of RPE cells with H_2_O_2_ 0.6 mM induced cell death (21 ± 3%), mainly by late apoptosis/necrosis (19 ± 1%). Co‐treatment with CIT 100 mM significantly reduced the percentage of total dead cells (10 ± 3%) and late apoptotic/necrotic cells (8 ± 3%). Cell death was also quantified by measurement of lactate dehydrogenase (LDH) activity in cell culture supernatants. As shown in Figure 4B, exposure of RPE cells to H_2_O_2_ 0.6 mM increased LDH release by 74% compared to the untreated cells (CTRL), whereas co‐treatment with CIT 100 mM decreased this release by 30% compared with the cells treated with H_2_O_2_ alone.

**FIGURE 5:**
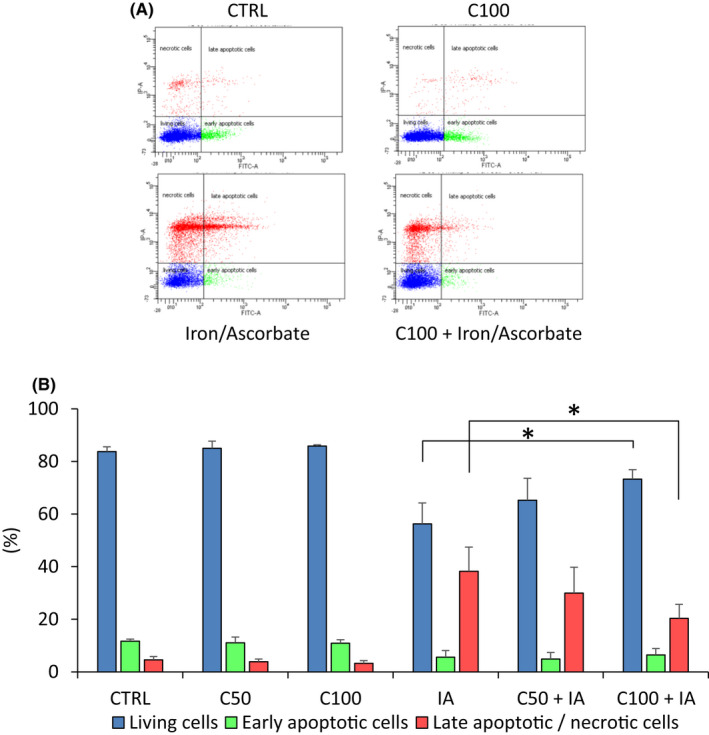
Effects of CIT on cell death in iron/ascorbate (I/A) stressed RPE cells. ARPE‐19 cells were pretreated with CIT 50 mM (C50) or 100 mM (C100) for 24 h and then exposed to I/A (7.5 mM/0.3 M) for 4 h. Cell death was quantified by flow cytometry using annexin V‐FITC (Ann) and propidium iodide (PI). The cell populations were identified as follows: living cells (Ann−, PI−), early apoptotic cells (Ann+, PI−) and late apoptotic/necrotic cells (Ann+, PI+). (A) Representative flow cytometry analysis showing living cells (Q1), early apoptotic cells (Q2) and late apoptotic (Q3)/necrotic cells (Q4). (B) The results are expressed as the percentage of total cells and presented as means ± SEM (*n* = 4 independent experiments). Exposure of RPE cells to iron/ascorbate increases cell death, mainly late apoptosis/necrosis, whereas pretreatment with CIT significantly reduces this increase. *p < 0.05 vs. treatment with I/A alone, *t*‐test. CIT, L‐Citrulline; RPE, retinal pigment epithelium

To examine the effect of CIT on I/A‐induced cell death, cell cultures were pretreated with CIT for 24 h and then exposed to I/A for 4 h, and cell death was quantified by flow cytometry. As shown in Figure 5, exposure of RPE cells to I/A 7.5 mM/0.3 M led to significant cell death (44%), mainly by late apoptosis/necrosis (38 ± 9%). A pretreatment of the cells with CIT 50 and 100 mM, before exposure to I/A, significantly reduced the percentage of total dead cells (35% and 27%, respectively) and specifically late apoptotic/necrotic cells (30 ± 10% and 20 ± 5%, respectively).

### 3.6 CIT attenuates H_2_O_2_‐induced IL‐8 secretion in RPE cells

**FIGURE 6:**
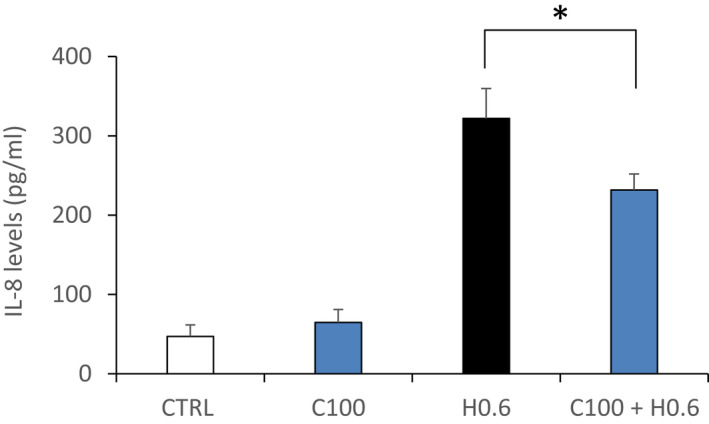
Effects of CIT on IL‐8 production in hydrogen peroxide (H_2_O_2_) stressed RPE cells. ARPE‐19 cells were treated with H_2_O_2_ 0.6 mM (H0.6) in the presence of CIT 100 mM (C100) for 4 h. Interleukin‐8 (IL‐8) released in the medium was determined by ELISA. The results are expressed as pg of IL‐8 per ml of medium. Data are presented as means ± SEM (*n* = 3 independent experiments). Treatment of RPE cells with H_2_O_2_ increases the level of IL‐8, whereas co‐treatment with CIT significantly reduces this increase. *p < 0.05 vs. H_2_O_2_‐exposed cells without CIT treatment, *t*‐test. CIT, L‐Citrulline; RPE, retinal pigment epithelium

We measured the level of the pro‐inflammatory cytokine IL‐8 in RPE cells treated with H_2_O_2_ in the presence of CIT. As shown in Figure 6, exposure of the cells to H_2_O_2_ 0.6 mM for 4 h led to significantly increased expression of IL‐8 by 6.8‐fold as compared to the untreated cells (CTRL). A co‐treatment with CIT 100 mM significantly decreased the level of IL‐8 by 30 ± 3% compared to cells treated with H_2_O_2_ alone.

## 4 DISCUSSION

In the work presented herein, we evaluated the effectiveness of CIT in protecting RPE cells from damage induced by oxidative stress. To our knowledge, we show for the first time that CIT supplementation is capable to protect RPE cells when challenged with toxic doses of H_2_O_2_ or iron/ascorbate. This is the first study describing the effects of CIT on oxidative stressed retinal cells.

In this study, we used H_2_O_2_ and iron/ascorbate to induce oxidative damage in RPE cells. H_2_O_2_ is widely used as a model of oxidative stress in RPE cells to mimic the pathogenesis of ARMD. This oxidant is increased in RPE during phagocytosis of shed photoreceptor outer segments^5^, ^6^ and during light irradiation of melanin in the RPE.^7^ H_2_O_2_ is also generated by the photo‐excited pigment lipofuscin accumulating during ageing in the RPE, and the accumulation of lipofuscin is strongly associated with ARMD.^8^ The iron/ascorbate system is commonly used in many studies to generate free radicals and lipid peroxidation.^33^, ^34^, ^35^, ^36^, ^37^ Iron levels increase in RPE during ageing, which may contribute to ARMD as an important source of oxidative stress.^9^, ^10^, ^11^ The increase of intracellular ferrous iron produces hydroxyl and lipid alkoxyl radicals through the Fenton reaction, which causes lipid peroxidation, membrane damage and cell death.^12^, ^13^

First, before testing the potential efficiency of a CIT treatment, we assessed its toxicity towards RPE cells. We observed that CIT alone did not affect the RPE viability at concentrations up to 100 mM, as determined by MTT assay. Thus, CIT safety was confirmed in cultured RPE cells, as it had already been evidenced in humans.^25^, ^28^

Then, we evaluated the protective effects of CIT against oxidative damage on RPE cell metabolic activity. We showed that CIT was effective against H_2_O_2_ in co‐treatment and against iron/ascorbate in pretreatment. Furthermore, the antioxidant effect of CIT has proved its efficiency by decreasing ROS production in RPE cells exposed to H_2_O_2_. This antioxidant effect could be attributed to the action of CIT as a scavenger of hydroxyl radicals produced from H_2_O_2_ or iron/ascorbate through the Fenton reaction, and to the activation of antioxidant/detoxifying enzymes including catalase. This is in agreement with the work of Akashi et al.^18^ who reported that CIT, present in high amounts in watermelon leaves (about 200 mM) which are resistant to stress induced by drought, was an effective scavenger of hydroxyl radicals. The authors showed that CIT at 50–400 mM was able to protect DNA and pyruvate kinase from oxidative damage, and that incubation of CIT with hydroxyl radicals produced from H_2_O_2_ resulted in reduction of the amount of CIT and in formation of secondary products. They calculated the constant rate for the reaction between CIT and hydroxyl radicals which was found to be 3.9.10^9^ M^−1^ s^−1^, demonstrating that CIT is one of the most potent scavengers. The half‐life of hydroxyl radicals for CIT was estimated to be significantly smaller than those for antioxidants ascorbate and glutathione (0.9, 1.9 and 17.5 ns, respectively). Therefore, the function of CIT as a hydroxyl radical scavenger may be more important than that of the classical antioxidants. In the same way, Ginguay et al.^38^ showed a protection of CIT in vitro on H_2_O_2_‐induced damage in human neuroblastoma SH‐SY5Y cells. The authors also reported a protective effect of CIT ex vivo on H_2_O_2_‐induced long‐term potential (LTP) impairment in hippocampal slices from young adult mice, and highlighted a beneficial effect of a CIT supplementation in vivo on age‐related LTP impairment in rats. They suggested that the antioxidant properties of CIT could result from its own oxidation by hydroxyl radicals produced from H_2_O_2_ through the Fenton reaction. Our results are also consistent with a study of Li et al.^39^ who evaluated the efficacy of CIT in vitro against oxidative stress in fish erythrocyte cells. The authors showed that CIT incubated in the presence of FeSO_4_/H_2_O_2_, used to generate hydroxyl radicals, was able to protect from oxidative damage, by decreasing ROS production and cell death and by increasing catalase, SOD and GPx activities.

Lipid peroxidation, a consequence of oxidative stress, plays an important role in the degeneration of RPE. As described above, the yellow‐brown fluorescent pigment lipofuscin accumulates in RPE with age and this aged accumulation has been associated with ARMD. Moreover, lipofuscin has been shown to produce ROS (singlet oxygen, superoxide anion and hydrogen peroxide) and to increase lipid peroxidation.^40^ In our study, exposure of RPE cells to H_2_O_2_ increased lipid peroxidation, while co‐treatment with CIT had a significant beneficial effect. On the contrary, incubation of RPE cells with iron/ascorbate also increased lipid peroxidation, whereas pretreatment with CIT limited this oxidation. This is in accordance with a work of Fu et al.,^19^ who evaluated the protective effects of CIT against renal ischaemia‐reperfusion injury in rats. The authors showed that CIT administered by gavage was able to decrease renal oxidative stress and to inhibit lipid peroxidation. Our findings are also in accordance with the study of Moinard et al.^21^ exploring the impact of CIT‐enriched diet in healthy aged rats. The authors found that CIT supplementation was able to lower the susceptibility to oxidation of lipoproteins (lag phase significantly higher and maximal concentration of conjugated diene significantly lower).

We also examined whether the protective effects of CIT on RPE cell metabolic activity was associated with an effect on cell death. The mechanism of RPE cell death in response to oxidative stress and in ARMD is debated in the literature. Most studies have implicated apoptosis as a principal process of cell death while others proposed necrosis as a major mechanism for RPE death.^41^, ^42^ In our study, treatment of RPE cultures with H_2_O_2_ 0.6 mM led to cell death, mainly by late apoptosis/necrosis. This is in agreement with the literature, which reports that H_2_O_2_ can trigger cell apoptosis when supplied at low concentrations and necrosis at higher concentrations.^43^, ^44^ Li et al.^45^ also reported that high concentration of H_2_O_2_ was able to cause RPE cell death with typical features of necrosis such as cell swelling, loss of plasma membrane integrity and nuclear condensation. They also reported that H_2_O_2_‐induced necrosis was a regulated process with cellular calcium overload as a critical step in the cell death program. In addition, Hanus et al.^41^ showed that features of apoptosis were not observed in RPE cells when exposed to H_2_O_2_. Instead, cardinal features of necrosis, such as rescue of cell death by RIP kinase inhibitors necrostatins, aggregation of the receptor‐interacting protein kinase 3, and change and breakdown of nuclear and plasma membrane permeability shown by PI staining and high mobility group proteins B1 release, were observed in the treated cells.^41^ In our work, co‐treatment of RPE cells with CIT and H_2_O_2_ led to a reduction of cell death (late apoptosis/necrosis) compared to cells treated with H_2_O_2_ alone. We also showed that exposure of RPE cells to the oxidant increased lactate dehydrogenase (LDH) release, whereas co‐treatment with CIT attenuated this release. The measurement of LDH activity is a marker of loss of plasma membrane integrity and thus of cell death by necrosis.

An increasing number of recent studies have reported that ferroptosis, a form of regulated necrosis characterized by iron accumulation and lipid peroxidation, is involved in the oxidative stress‐induced RPE cell death.^12^, ^46^ Retinal iron levels increase with age,^47^ and excessive iron accumulation is a source of free radical production in RPE.^11^ Moreover, iron levels in RPE have been found to be higher in ARMD patients,^10^, ^11^ suggesting that it may be implicated in the pathogenesis of the disease. Recently, a study has shown that intracellular iron can interact with bisretinoid lipofuscin in RPE to promote cell damage.^14^ In our study, exposure of RPE cultures to iron/ascorbate led to a significant cell death, mainly by late apoptosis/necrosis, and pretreatment with CIT significantly reduced this cell death. Our results are in agreement with the work of Fu et al.^19^ who examined the effects of CIT on renal ischaemia‐reperfusion injury in rats. Kidneys of ischaemic rats showed glomerular lesions and massive tubular epithelial cells necrosis or collapse, whereas pretreatment with CIT preserved the normal morphology of the kidneys.

Inflammation is also implicated in the molecular mechanisms of ARMD pathogenesis, leading to RPE damage. IL‐8, a pro‐inflammatory and pro‐angiogenic cytokine, is an important mediator of inflammation, and the increased expression of IL‐8 could explain, at least in part, the inflammatory events involved in ARMD.^17^ In the present work, we observed that treatment with H_2_O_2_ caused a significant production of IL‐8 by RPE cells. Our results are in agreement with the work of Fernandes et al.^17^ who have reported that oxidative stress induced by H_2_O_2_ stimulates IL‐8 production in RPE cells. The authors have also reported that photooxidation of A2E, the major component of lipofuscin, increases production of IL‐8. In our work, we showed that co‐treatment of RPE cells with CIT and H_2_O_2_ limited the production of IL‐8 induced by H_2_O_2_. The decrease in IL‐8 level could be explained by the direct antioxidant property of CIT on H_2_O_2_, thus reducing IL‐8 production. It could also be due to an effect of CIT on the activity of nuclear factors involved in the cytokine regulation. Indeed, IL‐8 is encoded on the CXCL8 gene whose transcription is regulated by repression of the CXCL8 promoter, transcriptional activation by inducible transcription factors and mRNA stabilization. Previous works have shown, in a model of cystic fibrosis airway cells, that H_2_O_2_ supplementation leads to oxidative stress and hyperacetylation at the NF‐kB site in the IL‐8 promoter conducting to IL‐8 protein expression.^48^ Thus, through its hydroxyl radical scavenging activity, CIT could have a direct action on IL‐8 expression. Furthermore, it has been reported, in intestinal ischaemia and reperfusion rat model, that oral CIT supplementation can act on the activity of transcription factor NF‐kB by decreasing the ratio of the phosphorylated to the total NF‐kB.^49^ Preclinical and clinical studies have also reported anti‐inflammatory effects of CIT. For instance, Breuillard et al.^50^ have evidenced anti‐inflammatory properties of CIT, which normalizes nitric oxide production variability by peritoneal macrophages, both in vitro and in vivo, in aged rats with endotoxin challenge. Van Vliet et al.^51^ have also reported in patients with chemotherapy‐induced mucosal barrier injury that plasma CIT was negatively correlated to plasma IL‐8 levels. Also, Luiking et al.^52^ have shown in patients with sepsis that C‐reactive protein was negatively correlated to plasma CIT concentration.

Finally, an important question is how CIT could have such effects? This could be related to the direct antioxidant potential of CIT,^18^, ^21^ to the activity of CIT on nuclear factors involved in the IL‐8 regulation,^48^, ^49^ and also to its capacity to generate nitric oxide as already observed.^23^ The last hypothesis could be related to the thermodynamic properties of CIT. We recently demonstrated that CIT was able to reallocate ATP consumption to muscle protein synthesis.^53^ To summarize, in stress situations (like in our conditions), there is a decrease in ATP/ADP ratio that leads to a decrease in Gibbs free energy of ATP hydrolysis. In such conditions, many reactions (requiring high levels energy in cells) are no longer possible and it may lead to cell death. By its thermodynamic action, CIT may decrease activation energies of one or several ATP (and GTP)‐consuming reactions involved in cell and, in fine, preserve cell from death.^54^ Alterations of the cellular energy dynamics with reduced ATP have been reported in H_2_O_2_‐treated ARPE‐19 cells^55^ and are classically observed during oxidative stress. Thus, we assume that the thermodynamic properties of CIT could also explain in part its protective effect.

In summary, our results evidence that CIT is capable to protect human RPE cells against H_2_O_2_‐ and iron/ascorbate‐induced damages: CIT improves cell metabolic activity, decreases ROS production, limits lipid peroxidation, reduces cell death and attenuates IL‐8 secretion. This suggests potential effects of CIT in the prevention or treatment of retinal diseases associated with oxidative stress, such as ARMD. Further studies will be necessary to examine in more details the mechanisms of action of the effective CIT against oxidative damage in RPE cells.

## CONFLICT OF INTEREST

C. Loï and C. Moinard are CITRAGE^®^ shareholders. The other authors do not have any conflict of interest.

## AUTHOR CONTRIBUTIONS

**Chervin Hassel:** Investigation (equal); Writing – original draft (equal). **Morgane Couchet:** Writing – original draft (equal). **Nathalie Jacquemot:** Formal analysis (equal); Investigation (equal); Visualization (equal). **Christelle Blavignac:** Formal analysis (equal); Investigation (equal). **Cécile Loï:** Writing – review & editing (equal). **Christophe Moinard:** Writing – review & editing (equal). **David Cia:** Conceptualization (lead); Formal analysis (equal); Funding acquisition (lead); Investigation (lead); Project administration (lead); Supervision (lead); Validation (lead); Visualization (equal); Writing – original draft (lead); Writing – review & editing (equal).

## Data Availability

The data that support the findings of this study are available from the corresponding author upon reasonable request.
